# Population-based incidence rates and increased risk of *EGFR* mutated non-small cell lung cancer in Māori and Pacifica in New Zealand

**DOI:** 10.1371/journal.pone.0251357

**Published:** 2021-05-07

**Authors:** Phyu Sin Aye, Mark James McKeage, Sandar Tin Tin, Prashannata Khwaounjoo, J Mark Elwood

**Affiliations:** 1 Epidemiology and Biostatistics, University of Auckland, Auckland, New Zealand; 2 Pharmacology and Clinical Pharmacology, University of Auckland, Auckland, New Zealand; 3 Auckland Cancer Society Research Centre, University of Auckland, Auckland, New Zealand; Osmania University, Hyderabad, India, INDIA

## Abstract

**Background:**

Non-squamous non-small cell lung cancer (NSCLC) patients with Epidermal Growth Factor Receptor (*EGFR*) mutation benefit from targeted treatments. Previous studies reported *EGFR* mutation-positive proportions among tested non-squamous NSCLC patients. However, incidence rates and population risk of *EGFR* mutation-positive and *EGFR* mutation-negative non-squamous NSCLC have not been assessed. This study therefore aimed to estimate the population-based incidence rates of *EGFR* mutation-positive and *EGFR* mutation-negative non-squamous NSCLC in different population groups defined by sex, ethnic group and smoking status.

**Methods:**

This study included data from all non-squamous NSCLC patients diagnosed in northern New Zealand between 1/02/2010 and 31/07/2017 (N = 3815), obtained from a population-based cancer registry. Age-specific incidence rates, WHO age-standardised rates (ASRs) and rates adjusted for incomplete testing were calculated for *EGFR* mutation-positive and *EGFR* mutation-negative diseases for the study cohort as a whole and subgroups of patients.

**Results:**

Among 3815 patients, 45% were tested for *EGFR* mutations; 22.5% of those tested were *EGFR* mutation-positive. The ASR of *EGFR* mutation-positive NSCLC was 5.05 (95%CI 4.71–5.39) per 100,000 person-years. ASRs for *EGFR* mutation-positive NSCLC were higher for females than males: standardised incidence ratio (SIR) 1.50 (1.31–1.73); higher for Pacifica, Asians and Māori compared with New Zealand Europeans: SIRs 3.47 (2.48–4.85), 3.35 (2.62–4.28), and 2.02 (1.43–2.87), respectively; and, only slightly increased in ever-smokers compared with never-smokers: SIR 1.25 (1.02–1.53). In contrast, the ASR of *EGFR* mutation-negative NSCLC was 17.39 (16.75–18.02) per 100,000 person-years, showing a strong association with smoking; was higher for men; highest for Māori, followed by Pacifica and then New Zealand Europeans, and lowest for Asians. When corrected for incomplete testing, SIRs by sex, ethnicity and smoking, for both diseases, remained similar to those based on tested patients.

**Conclusion:**

The population risk of *EGFR* mutation-positive NSCLC was significantly higher for Māori and Pacifica compared with New Zealand Europeans.

## Introduction

Lung cancer was the most common cancer for both sexes globally, accounting for 11.6% of total cancer incidence in 2018 [1]. In New Zealand, it contributed 8.4% of total cancer incidence in 2017, showing an age-standardised rate of 27.8 cases per 100,000 population [2]. Lung cancer, according to pathology, can be classified into two main groups: non-small cell lung cancer (NSCLC) and small cell lung cancer, accounting for 85% and 15%, respectively [3]. Squamous NSCLCs contribute about 25–30% of all lung cancers; the remaining non-squamous NSCLCs comprise adenocarcinoma, large-cell carcinoma, and NSCLC not otherwise specified (NOS). Non-squamous NSCLCs may harbour targetable mutations which define different subtypes of lung cancer with different aetiologies, molecular pathology, personalised treatment pathways and disease prognosis [4].

A significant advance in personalised treatment for NSCLC patients was the identification of lung cancers with Epidermal Growth Factor Receptor (*EGFR*) gene mutations [5], which benefit from targeted treatments with EGFR tyrosine kinase inhibitors (TKIs) such as gefitinib, erlotinib and osimertinib [6,7], with significantly prolonged progression-free survival compared to standard chemotherapy [8]. With the approval of the use of EGFR-TKIs in *EGFR* mutation-positive lung cancer patients, *EGFR* mutation testing is generally recommended to non-squamous NSCLC patients [9–13].

Most previous studies of *EGFR* mutation-positive lung cancer have reported the proportion of patients with *EGFR* mutations among the tested patients. Such *EGFR* mutation-positive proportions vary widely from 10% to 51% in non-squamous NSCLC patients depending on sex, ethnicity and smoking status [14–16]. A systematic review covering 151 worldwide studies published up to 2014 observed that the *EGFR* mutation-positive proportion was reported as highest in the Asia-Pacific region (China, Hong Kong, Japan, Korea and Taiwan) and lowest in Oceania (Australia)– 47% versus 12% [16]. Specifically, the *EGFR* mutation-positive proportions were 60% in women and 37% in men, and 64% in never-smokers and 33% in ever-smokers as in the Asia-Pacific subgroup [16].

Despite the various reports on *EGFR* mutation-positive proportions, it is difficult to estimate the actual disease burden as most studies are not population-based, so little is known about the incidence rates of *EGFR* mutation-positive and negative non-squamous NSCLC in the general population and in subgroups. This population-based study therefore aimed to estimate the population-based incidence rates of *EGFR* mutation-positive and *EGFR* mutation-negative non-squamous NSCLC in different population groups defined by age, sex, ethnic group—especially the indigenous Māori people and Pacific peoples, and smoking status, based on northern New Zealand data.

## Materials and methods

### Study population

This study used the data of all patients who presented with non-squamous NSCLC in northern New Zealand, which comprises Northland, Waitemata, Auckland and Counties Manukau, contributing approximately 40% of New Zealand population, between 1 February 2010 and 31 July 2017 (N = 3815). We previously reported on the *EGFR* mutation testing [17,18] and developed a predictive model to estimate the *EGFR* mutation status [19] using this study cohort. In this study, we expanded the analysis to include population data to estimate the population-based *EGFR* mutation-positive and *EGFR* mutation-negative non-squamous NSCLC incidence.

Eligible patients were identified from the New Zealand Cancer Registry (NZCR), which is a well-established legally-mandated population-based cancer registry that registers all primary cancers diagnosed in New Zealand, excluding squamous and basal cell skin cancers [20]. Information on the following patient background characteristics was extracted from the NZCR: National Health Index (NHI) number, district health board (DHB) of residence, date of birth, date of diagnosis, gender, ethnicity and smoking status. These data were linked, using the NHIs, to individual patient medical records to obtain more information on smoking status, and to TestSafe to obtain information on *EGFR* mutation status. TestSafe is a clinical information sharing service in northern New Zealand that compiles the laboratory and radiology results and reports from DHB and community facilities [21].

*EGFR* mutations were detected by the Roche Cobas^®^ real-time PCR [22] or Agena MassARRAY OncoFOCUS^™^ [23] tests, which were validated previously [24]. We categorised all the diagnosed *EGFR* mutations as *EGFR*(+) in the analysis regardless of their sensitivity to EGFR-TKIs [25]; these included G719X and E709A in exon 18, exon 19 deletions, R776C, S768I, T790M and insertions in exon 20, L858R and L861Q in exon 21, detected alone or in combination (S3 Table). A majority of EGFR mutations (exon 19 deletion and exon 21 L858R; 80.5%) are sensitive to EGFR-TKIs (S3 Table). With rapidly advancing research, newer generations of EGFR-TKIs are emerging that can target those EGFR mutations currently known to be resistant. Therefore, in our current study, we included all EGFR mutations regardless of the sensitivity to EGFR-TKIs.

The ethical approval for this research was obtained from the New Zealand Government Ministry of Health Northern B Health and Disability Ethics Committee (reference: 13/NTB/165/AM02). This research used routinely collected data and did not involve direct contact with patients. The human participants in this retrospective study were not required to give informed consent because informed consent was considered impractical and undesirable by both the researchers and by the ethics committee and governance groups who approved the study. Individual patient-level data were provided from various sources to the researchers without anonymization. These data were then compiled into a study database that included several direct and indirect identifiers. The ethical and legal requirements of the Ministry of Health for maintaining confidentiality and privacy of the study participants were met by limiting access to the study database to the healthcare professionals and research staff who were directly involved in the project and sharing only aggregate and deidentified data.

### Data analysis

The analyses were based on the total of 3815 non-squamous NSCLC patients, limited to 3776 in analyses by ethnicity, and to 1855 in analyses by smoking status due to missing data. The smoking status was grouped into never-smokers and ever-smokers; the latter comprises current smokers and former smokers. The ethnic groups were categorised as New Zealand European, Māori, Pacific and Asian according to the New Zealand national collections [26].

Within each sex, ethnicity, smoking status group and 10-year age group, the proportion of EGFR mutation-positive non-squamous NSCLC among tested patients was multiplied by the annual numbers of non-squamous NSCLC to estimate the annual numbers of EGFR mutation-positive cases. These numbers of EGFR mutation-positive cases were then divided by the appropriate northern New Zealand population using the 2013 New Zealand census data (S2 Table) to estimate population-based incidence rates, reported annual numbers per 100,000 population (i.e. per 100,000 person-years).

The incidence rates were presented as crude rates, age-specific rates and age-standardised rates (ASRs) using the WHO world standard population [27] (S1 Table) based on the tested patient proportions. The incidence rates were also corrected for incomplete testing using the formula published in our previous research article [18]. The ASRs between groups were compared by means of Standardised Incidence Ratios (SIRs). The definitions of the estimates and formulas are depicted below. The results were reported in groups specified by gender, ethnicity and smoking status. The data analyses were conducted using Stata 16 and Microsoft Excel.

***Tested proportions (%)***: are the numbers of those tested for *EGFR* mutation among all non-squamous NSCLC cases, shown in per cent.

***EGFR mutation-positive proportions (%)***: are the number of *EGFR* mutation-positive patients divided by the total number of tested patients, shown in per cent.

***Crude and age-specific EGFR mutation-positive incidence rates***: are the annual number of *EGFR* mutation-positive non-squamous NSCLC per 100,000 population, where crude rates are overall rates and the rates in each age group are age-specific rates. The resident population was obtained from the 2013 New Zealand census data [28].

***Age-standardised EGFR mutation-positive incidence rates (ASRs)***: are the *EGFR* mutation-positive non-squamous NSCLC incidence rates that are age-standardised using the WHO world standard population [27] (S1 Table). It was calculated as the sum of the product of age-specific *EGFR* mutation-positive incidence and WHO standard population proportions in the respective age groups, divided by the total WHO standard population.

***Standardised incidence ratios (SIRs)***: are the ratios of two age-standardised incidence rates (ASRs). The 95% confidence intervals (CIs) of *ASRs* and *SIRs* were calculated using the methods published by Boyle & Parkin (1991) [29].

***EGFR mutation-negative incidence rates***: were obtained by applying the same concepts of incidence rate calculations for the *EGFR* mutation-positive disease to the *EGFR* mutation-negative disease. However, we only presented age-standardised incidence rates (ASRs) for *EGFR* mutation-negative disease, without crude rates, in this study.

***Uncorrected incidence rates*** *(crude or ASRs)*: are those calculated based on the actual tested proportions.

***Corrected incidence rates*** *(crude or ASRs)*: refer to those estimated based on 100% testing (i.e. if all non-squamous NSCLC patients were tested for *EGFR* mutation) using the following formula published in our previous study [18].

$$m_{1}=e^{\text{ln}\left(m\right)–0.994\times (1-t)},$$

where

m_1_ = estimated proportion of *EGFR* mutation-positive cases if all patients were tested,

m = observed proportion of *EGFR* mutation-positive cases in tested patients, and

t = proportion of patients tested.

## Results

This study included 3815 non-squamous NSCLC patients in total (Table 1). The patients were predominantly 60–79 years old (2237/3815, 58.6%), females (1950/3815, 51.1%), of New Zealand European ethnic group (2335/3776, 61.8%), and ever-smokers (1380/1855, 74.4%). About 45% (n = 1709) of the total 3815 patients had *EGFR* mutation testing. Specifically, *EGFR* mutations was more often tested for younger patients– 15–39 years (82.1%) and 40–59 years (57%); females (49.1%); and Asians (61.3%). Smoking data were mostly available for tested patients; thus, a majority of patients in both smoking groups were tested patients (ever-smokers 90.8% and never-smokers 89.7%).

**Table 1:** Numbers of non-squamous NSCLC patients, patients tested for *EGFR* mutations, and *EGFR* mutation-positive patients shown by age, gender, ethnicity and smoking status in northern New Zealand, between 1 February 2010 and 31 July 2017.

	Non-squamous NSCLC	Tested	*EGFR*(+)
	N	N (%)	N (%) among tested
Overall	3815	1709 (44.8)	384 (22.5)
Age			
15–29	6	5 (83.3)	1 (20.0)
30–39	22	18 (81.8)	4 (22.2)
40–49	152	92 (60.5)	34 (37.0)
50–59	513	287 (55.9)	66 (23.0)
60–69	1068	538 (50.4)	99 (18.4)
70–79	1169	575 (49.2)	135 (23.5)
80+	885	194 (21.9)	45 (23.2)
Gender			
Male	1865	752 (40.3)	123 (16.4)
Female	1950	957 (49.1)	261 (27.3)
Ethnicity			
NZ European	2335	1006 (43.1)	166 (16.5)
Māori	643	248 (38.6)	27 (10.9)
Pacific	392	183 (46.7)	53 (29.0)
Asian	406	249 (61.3)	129 (51.8)
Smoking status			
Never-smoker	475	426 (89.7)	212 (49.8)
Ever-smoker	1380	1253 (90.8)	166 (13.2)

Total 3815 non-squamous NSCLC; limited to 3776 in analyses by ethnicity; and limited to 1855 in analyses by smoking status due to missing data. Ever-smokers comprise current smokers and former smokers.

### *EGFR* mutation-positive proportions

Among those tested, 22.5% (384 patients) were *EGFR* mutation-positive, being higher in females (27.3%) and never-smokers (49.8%) compared to their counterparts (Table 1). Among ethnic groups, Asians had the highest proportion (51.8%), followed by Pacifica (29%) and New Zealand Europeans (16.5%), with Māori having the lowest proportion (10.9%). The *EGFR* mutation-positive proportions varied with age, ranging from 18.4% in 60–69 years to 37% in 40–49 years (Table 1). However, it was unclear from these *EGFR* mutation-positive proportion values how the trends relate to the incidence rates of *EGFR* mutation-positive or negative disease. For example, Māori may have had a lower proportion of *EGFR* mutation-positive cancers because of a higher incidence of *EGFR* mutation-negative disease, a lower incidence of *EGFR* mutation-positive disease or both.

### Total and age-specific incidence rates by *EGFR* mutation status

Incidence rates were then calculated separately for *EGFR* mutation-positive and *EGFR* negative disease based on the tested proportions.

**Fig 1:**
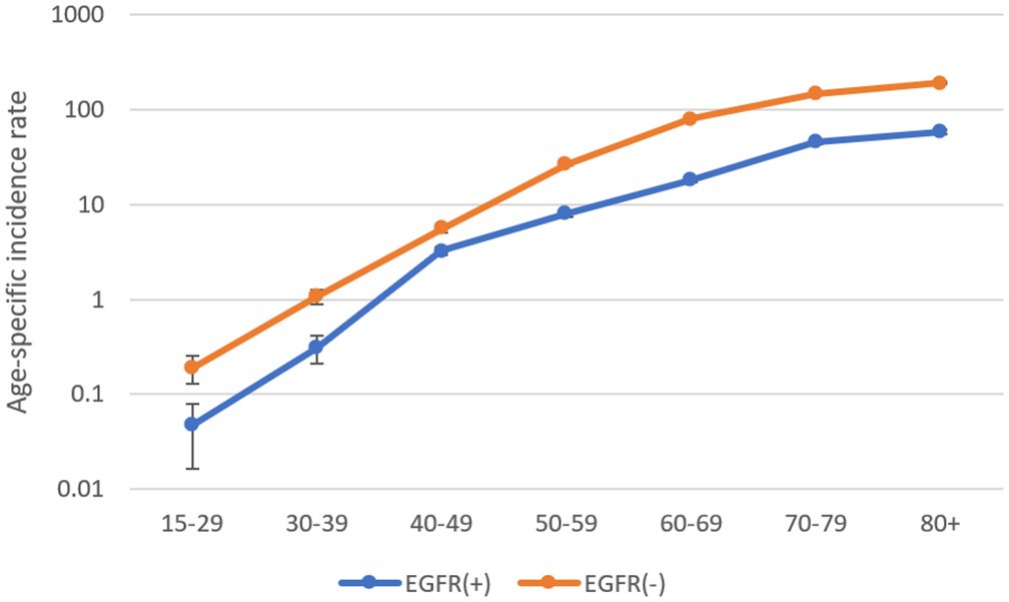
Age-specific incidence rates (cases per 100,000 person-years) of non-squamous non-small cell lung cancer by *EGFR* mutation status, estimated based on the *EGFR* mutation tested proportions, shown in age groups. The vertical error bars represent 95% confidence intervals of incidence rates.

The crude all-ages incidence rate of *EGFR* mutation-positive disease was 9.14 (95% Confidence Interval (95%CI) 8.9–9.4) per 100,000 person-years. The age-specific rates increased with age, ranging from 0.05 (95%CI 0.02–0.08) in the youngest 15–29 years group to 58.56 (95%CI 55.64–61.49) per 100,000 person-years in the oldest 80+ years group (Fig 1).

The crude all-ages incidence rate of *EGFR* mutation-negative disease was 31.6 (95%CI 31.2–32.0) per 100,000 person-years. The age-specific rates showed a similar increase with age as mutation-positive disease, ranging from 0.19 (95%CI 0.13–0.25) in the youngest 15–29 years group to 193.9 (95%CI 188.6–199.23) per 100,000 person-years in the oldest 80+ years group (Fig 1).

### Age-standardised incidence rates by *EGFR* mutation status

To compare rates of disease between different groups, age-standardised incidence rates were calculated. The WHO standard population has larger numbers in younger age groups and fewer in older age groups compared to the population of a developed country like New Zealand (S1 & S2 Tables). Thus, for lung cancer where the incidence rates are much higher in older age groups, the WHO age-standardised incidence rates are considerably lower than actual incidence rates. The age distribution also varies by population subgroups. For example, Maori and Pacifica has younger age distribution compared to New Zealand Europeans. Therefore, differences between crude rates and ASR were more pronounced for New Zealand Europeans.

**Fig 2:**
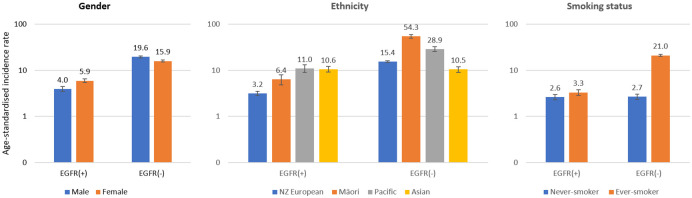
Age standardised incidence rates of non-squamous NSCLC (based on the *EGFR* mutation tested proportions) in terms of *EGFR* mutation status categorised by gender, ethnicity and smoking status. Age standardised rates represent cases per 100,000 person-years and are based on WHO world standard population. Analysis based on total 3815 non-squamous NSCLC; limited to 3776 in analyses by ethnicity; and limited to 1855 in analyses by smoking status due to missing data. Ever-smokers comprised current smokers and former smokers. The vertical error bars represent 95% confidence intervals of incidence rates.

Based on tested proportions, the age-standardised incidence rate (ASR) of total non-squamous NSCLC was 22.4 per 100,000 person-years. The ASR for *EGFR* mutation-positive NSCLC was 5.05 (95%CI 4.71–5.39) per 100,000 person-years (Table 2). The ASR for *EGFR* mutation-positive NSCLC was higher for females than males: standardised incidence ratio (SIR) 1.50 (95%CI 1.31–1.73). Incidence rates were higher for Pacifica, Asians and Māori compared with New Zealand Europeans: SIR for Pacifica 3.47 (95%CI 2.48–4.85), Asians 3.35 (95%CI 2.62–4.28), Māori 2.02 (95%CI 1.43–2.87). The ASR of *EGFR* mutation-positive NSCLC was only slightly increased in ever-smokers compared with never-smokers: SIR 1.25 (95%CI 1.02–1.53) (Table 2, Fig 2).

**Table 2:** Incidence rates of non-squamous non-small cell lung cancer by *EGFR* mutation status, showing the estimates based on the *EGFR* mutation tested proportions.

	*EGFR*(+) crude incidence (95% CI)	Overall NSCLC ASR (95%CI)	*EGFR*(+) proportion	*EGFR*(+)		*EGFR*(-)	
				ASR (95%CI)	SIR (95%CI)	ASR (95%CI)	SIR (95%CI)
Overall	9.14 (8.92–9.36)	22.44 (21.72–23.16)	22.5	5.05 (4.71–5.39)	-	17.39 (16.75–18.02)	-
Gender							
Male	7.00 (6.72–7.28)	23.56 (22.48–24.63)	16.4	3.95 (3.51–4.39)	1	19.61 (18.63–20.59)	1
Female	10.79 (10.46–11.13)	21.81 (20.83–22.79)	27.3	5.93 (5.42–6.45)	1.50 (1.31–1.73)	15.88 (15.04–16.72)	0.81 (0.75–0.87)
Ethnicity							
NZ European	7.54 (7.28–7.81)	18.54 (17.77–19.32)	16.5	3.16 (2.84–3.48)	1	15.38 (14.68–16.09)	1
Māori	7.13 (6.51–7.75)	60.73 (55.89–65.58)	10.9	6.40 (4.83–7.97)	2.02 (1.43–2.87)	54.34 (49.75–58.92)	3.53 (3.04–4.11)
Pacific	11.32 (10.54–12.09)	39.84 (35.84–43.84)	29.0	10.95 (8.88–13.03)	3.47 (2.48–4.85)	28.88 (25.47–32.30)	1.88 (1.60–2.21)
Asian	11.11 (10.56–11.66)	21.04 (18.94–23.15)	51.8	10.57 (9.10–12.05)	3.35 (2.62–4.28)	10.47 (8.96–11.97)	0.68 (0.60–0.78)
Smoking status							
Never-smoker	4.23 (4.03–4.43)	5.30 (4.82–5.78)	49.8	2.64 (2.30–2.98)	1	2.66 (2.32–3.00)	1
Ever-smoker	6.48 (6.14–6.82)	24.35 (23.06–25.64)	13.2	3.31 (2.82–3.79)	1.25 (1.02–1.53)	21.04 (19.84–22.24)	7.92 (6.88–9.11)

ASR = Age-standardised incidence rates, cases per 100,000 person-years; SIR = standardised incidence ratio. Age standardised rates are based on WHO world standard population. Analysis is based on total 3815 non-squamous NSCLC; limited to 3776 in analyses by ethnicity; and limited to 1855 in analyses by smoking status due to missing data. Ever-smokers comprise current smokers and former smokers.

*EGFR* mutation-negative NSCLC was more common, showing an ASR of 17.4 (95%CI 16.75–18.02) per 100,000 person-years, based on the tested proportions (Table 2). In contrast to the *EGFR* mutation-positive disease, the ASR of *EGFR* mutation-negative NSCLC was lower in females than males: SIR 0.8 (95%CI 0.75–0.87); it was lower in Asians compared with New Zealand Europeans: SIR 0.68 (95%CI 0.6–0.78), but much higher in Māori: SIR 3.53 (95%CI 3.04–4.11) and higher in Pacifica: SIR 1.88 (95%CI 1.6–2.2). These ASR trends of *EGFR* mutation-negative lung cancer corresponded to the proportions of ever-smokers in those ethnic subgroups, except for Pacifica versus New Zealand Europeans (S2 Table). The ASR was much higher in ever-smokers compared with never-smokers: SIR 7.9 (95%CI 6.88–9.11) (Table 2, Fig 2).

To assess consistency over time, SIR’s by sex, ethnicity and smoking were assessed separately for the earlier period 2010–13 (n = 1864) and for the later period 2014–17 (n = 1951). The results were not substantially changed and all confidence limits overlapped; the SIRs for the whole time period presented here are similar to those restricted to the later time period.

### *EGFR* mutation-specific incidence rates corrected for incomplete testing

Incomplete testing may bias estimates of disease incidence within a given population [18]. When corrected for incomplete testing, the corrected *EGFR* mutation-positive incidence rates were generally somewhat lower compared to the respective uncorrected rates (Table 3). The age-specific incidence rates of *EGFR* mutation-positive NSCLC increased with age up to 70–79 years: 27.2 (95%CI 25.7–28.7) per 100,000 person-years (S1 Fig). The standardised incidence ratios by sex, ethnicity and smoking, for both *EGFR* mutation-positive and *EGFR* mutation-negative diseases, were similar (and in no instance significantly different) to the ratios of uncorrected rates already shown (Table 3).

**Table 3:** Incidence rates of non-squamous non-small cell lung cancer by *EGFR* mutation status, showing the estimates for 100% testing.

	*EGFR*(+) crude incidence (95%CI)	Overall NSCLC ASR (95%CI)	*EGFR*(+) proportion	*EGFR*(+)		*EGFR*(-)	
				ASR (95%CI)	SIR (95%CI)	ASR (95%CI)	SIR (95%CI)
Overall	5.33 (5.16–5.50)	22.44 (21.72–23.16)	13.0	3.00 (2.74–3.27)	-	19.43 (18.77–20.10)	-
Gender							
Male	3.86 (3.65–4.07)	23.56 (22.48–24.63)	9.0	2.20 (1.87–2.53)	1	21.36 (20.34–22.38)	1
Female	6.59 (6.33–6.85)	21.81 (20.83–22.79)	16.4	3.71 (3.30–4.12)	1.69 (1.41–2.02)	18.10 (17.21–18.99)	0.85 (0.79–0.91)
Ethnicity							
NZ European	4.22 (4.02–4.42)	18.54 (17.77–19.32)	9.4	1.84 (1.59–2.08)	1	16.70 (15.97–17.44)	1
Māori	3.97 (3.51–4.44)	60.73 (55.89–65.58)	5.9	3.47 (2.33–4.61)	1.89 (1.20–2.97)	57.26 (52.56–61.97)	3.43 (2.97–3.96)
Pacific	6.81 (6.21–7.41)	39.84 (35.84–43.84)	17.0	6.46 (4.88–8.03)	3.51 (2.28–5.42)	33.38 (29.71–37.06)	2.00 (1.71–2.33)
Asian	7.65 (7.19–8.11)	21.04 (18.94–23.15)	35.3	7.17 (5.97–8.37)	3.90 (2.85–5.34)	13.87 (12.14–15.60)	0.83 (0.73–0.94)
Smoking status							
Never-smoker	3.82 (3.63–4.00)	5.30 (4.82–5.78)	44.9	2.38 (2.06–2.70)	1	2.92 (2.56–3.28)	1
Ever-smoker	5.89 (5.57–6.22)	24.35 (23.06–25.64)	12.1	3.00 (2.54–3.46)	1.26 (1.02–1.55)	21.35 (20.14–22.56)	7.31 (6.38–8.38)

ASR = age-standardised incidence rates, cases per 100,000 person-years; SIR = standardised incidence ratio. Age standardised rates are based on WHO world standard population. Analysis is based on total 3815 non-squamous NSCLC; limited to 3776 in analyses by ethnicity; and limited to 1855 in analyses by smoking status due to missing data. Ever-smokers comprise current smokers and former smokers.

## Discussion

We estimated the population-based incidence rates of *EGFR* mutation-positive and *EGFR* mutation-negative non-squamous NSCLC based on all 3815 patients registered over 7.5 years. The age-standardised incidence rate (ASR) of non-squamous NSCLC was 22.4 cases per 100,000 person-years overall, 5.1 and 17.4 per 100,000 person-years for *EGFR*-positive and -negative disease, respectively, based on the tested proportions. The age-standardised incidence rates of *EGFR* mutation-positive disease were higher in women (SIR 1.5); highest in Pacific and Asian populations (SIRs 3.5, 3.4), followed by Māori (SIR 2.0), and lowest in New Zealand Europeans, all these associations being statistically significant. The ASR of *EGFR* mutation-positive disease was only slightly higher in ever-smokers than never-smokers (SIR 1.25). These associations were not influenced by effects in the earlier time period where testing was less frequent.

This study revealed that assessing the risk of *EGFR* mutation-positive lung cancer just by *EGFR* mutation-positive proportions in tested patients [14–16], ignoring underlying population incidence, can be misleading. Based only on the *EGFR* mutation-positive proportions, it appeared that being Māori was of lower risk and being Pacific was of higher risk but not as much as Asians, compared with New Zealand Europeans. On the contrary, the population-based incidence rates showed that Māori had approximately two times increased risk, and Pacifica and Asians had approximately 3.5 times increased risk for *EGFR* mutation-positive lung cancer compared with New Zealand Europeans.

The age-standardised incidence rates of *EGFR* mutation-positive NSCLC were close to or higher than those of many common cancers in New Zealand, such as cancers of stomach (5.3 cases per 100,000 population), brain (5.4), thyroid (5.8), cervix (6.1), ovary (6.6), testis (7.5), kidney (7.9), and leukaemia (10.2) based on 2017 diagnoses [2].

This study showed contrasting patterns of incidence rates between *EGFR* mutation-positive and negative non-squamous NSCLC. It suggests that the two diseases have different risk factors, in addition to having different management pathways. The majority group, with *EGFR* mutation-negative accounting for 77.5% of the total, was strongly associated with smoking, more common in men than women, and had the highest incidence in Māori, followed by Pacific peoples, the New Zealand European population, and then was lowest in people of Asian ethnicity. Most of these findings of *EGFR* mutation-negative group contradict those of *EGFR* mutation-positive group. These results are generally consistent with those of major reviews, which concluded that *EGFR* mutations and smoking had independent effects on lung cancer, and that *EGFR* mutations were more common in Asians, females, and never-smokers [16,30–33]. Smoking is a predominant risk factor of lung cancer; yet, lung cancer incidence in never-smokers is also evident [34]. Studies suggested that persistent differences in lung cancer incidence among different ethnic populations were not entirely explained by variations in smoking history [35–37], for which, *EGFR* mutation-positive lung cancer exemplifies [38]. Some studies claimed that interethnic genetic variations may play a role in *EGFR* mutation positivity [39,40]. Current understanding of the aetiology of lung cancer, and health policies and practices for its prevention and early detection, are based almost solely on risk factors for smoking-related lung cancer. A better understanding of the risk factors for *EGFR* mutation-positive lung cancer will be required to identify its causes and to develop public health strategies for its prevention and early detection.

Every population-based study of *EGFR* mutation testing has shown incomplete testing [41]. Evidence showed that testing tends to be selective due to multiple reasons, including limited testing facilities, high costs and insufficient tissue samples [17,18,41–43]. Patients with a higher chance of *EGFR* mutation positivity were therefore more likely to be offered testing, resulting in high *EGFR* mutation-positive proportions in the tested proportion. This was seen in this study. We and others have previously shown that as the proportions of all NSCLC cases tested has increased over time, the reported *EGFR* mutation-positive proportions have decreased [17,18]. We showed that in this northern New Zealand area, the *EGFR* mutation-positive proportions decreased from 43.8% to 16.8% in parallel with increased testing rates from 3.7% to 64.6% over the period of 2010–2014 [17,18]. If testing were complete rather than selective, the true proportions of mutations would be lower. We addressed this issue to reflect the true population incidence by estimating the corrected incidence rates, using a nonlinear regression equation, which assumes a decrease in *EGFR* mutation-positive proportion with an increase in testing, derived from our previous work [18]. This changed the estimated incidence rates substantially, but importantly, made little difference to the associations with sex, ethnicity and smoking that we reported. The largest effect was in the SIR for Asians for *EGFR* mutation-positive disease, increased by the correction for incomplete testing.

The key advantage of our study was the use of a large population-based dataset covering 7.5 years, obtained from the robust national data sources. This study was also the first to report the incidence of *EGFR* mutation-specific lung cancer in Māori and Pacifica, the populations of interest in New Zealand, revealing that these populations had increased risk of developing *EGFR* mutation-positive lung cancer, as seen for lung cancer overall [44–47]. Our study also had limitations. This study used the retrospective data and therefore the analyses were limited to the information that had been collected. Smoking data was missing for a significant proportion of patients, particularly those who were untested for *EGFR* mutation. It resulted in a smaller room for correction for incomplete testing in estimating the incidence rates by smoking status. More detailed information, for example, pack-years, which is the product of the number of cigarette packs smoked per day and the number of years smoked [48], may be useful for future research as smoking is a major risk factor [49] and is also related to *EGFR* mutation positivity [14]. In this analysis of population-based incidence rates, the available numbers did not permit the results for each factor to be adjusted for other factors, but in the previous analysis, we have used multivariable methods and demonstrated that the effects of gender, ethnicity, and smoking status are independent, and indeed can be combined to predict the mutation status of the cancer [19]. The estimation of the incidence of *EGFR* mutation-specific NSCLC can be improved as the testing becomes more complete in the future.

## Conclusions

The population-based incidence rates provide a more complete assessment of the risk of *EGFR* mutation-positive lung cancer than do the *EGFR* mutation-positive proportions. The ASRs of *EGFR* mutation-positive NSCLC were about 3.5 folds higher for Pacifica and Asians, and two folds higher for Māori compared with New Zealand Europeans.

## Supporting information

S1 Fig Age-specific incidence rates (cases per 100,000 person-years) of non-squamous non-small cell lung cancer by *EGFR* mutation status estimated for 100% testing, shown in age groups. The vertical error bars represent 95% confidence intervals of incidence rates.
(DOCX)

S2 Fig Age standardised incidence rates (estimated for 100% testing) of non-squamous NSCLC in terms of *EGFR* mutation status categorised by gender, ethnicity and smoking status. Age standardised rates represent cases per 100,000 person-years and are based on WHO world standard population. Analysis based on total 3815 non-squamous NSCLC; limited to 3776 in analyses by ethnicity; and limited to 1855 in analyses by smoking status due to missing data. Ever-smokers comprised current smokers and former smokers. The vertical error bars represent 95% confidence intervals of incidence rates.
(DOCX)

S1 Table WHO world standard population. (DOCX)

S2 Table Numbers of resident population, shown by smoking status, based on 2013 New Zealand census data. (DOCX)

S3 Table Different types of *EGFR* mutation by numbers of patients. (DOCX)
