# Supplementary material for: Population-based incidence rates and increased risk of EGFR mutated non-small cell lung cancer in Māori and Pacifica in New Zealand
Source: PLoS One. 2021 May 7;16(5):e0251357. doi: 10.1371/journal.pone.0251357 (PMC8104366; doi:10.1371/journal.pone.0251357)
Supplement: S1 Fig — The vertical error bars represent 95% confidence intervals of incidence rates. (DOCX) [file pone.0251357.s001.docx]

Fig S1. Age-specific incidence rates (cases per 100,000 person-years) of non-squamous non-small cell lung cancer by *EGFR* mutation status estimated for 100% testing, shown in age groups. The vertical error bars represent 95% confidence intervals of incidence rates.
