# Supplementary material for: Population-based incidence rates and increased risk of EGFR mutated non-small cell lung cancer in Māori and Pacifica in New Zealand
Source: PLoS One. 2021 May 7;16(5):e0251357. doi: 10.1371/journal.pone.0251357 (PMC8104366; doi:10.1371/journal.pone.0251357)
Supplement: S2 Fig — Age standardised rates represent cases per 100,000 person-years and are based on WHO world standard population. Analysis based on total 3815 non-squamous NSCLC; limited to 3776 in analyses by ethnicity; and limited to 1855 in analyses by smoking status due to missing data. Ever-smokers comprised current smokers and former smokers. The vertical error bars represent 95% confidence intervals of incidence rates. (DOCX) [file pone.0251357.s002.docx]

Fig S2. Age standardised incidence rates (estimated for 100% testing) of non-squamous NSCLC in terms of *EGFR* mutation status categorised by gender, ethnicity and smoking status. Age standardised rates represent cases per 100,000 person-years and are based on WHO world standard population. Analysis based on total 3815 non-squamous NSCLC; limited to 3776 in analyses by ethnicity; and limited to 1855 in analyses by smoking status due to missing data. Ever-smokers comprised current smokers and former smokers. The vertical error bars represent 95% confidence intervals of incidence rates.
