# Supplementary material for: Population-based incidence rates and increased risk of EGFR mutated non-small cell lung cancer in Māori and Pacifica in New Zealand
Source: PLoS One. 2021 May 7;16(5):e0251357. doi: 10.1371/journal.pone.0251357 (PMC8104366; doi:10.1371/journal.pone.0251357)
Supplement: S1 Table — (DOCX) [file pone.0251357.s003.docx]

Table S1. WHO world standard population

| Age group | Population |
| --- | --- |
| 15-29 | 246200 |
| 30-39 | 147600 |
| 40-49 | 126300 |
| 50-59 | 99200 |
| 60-69 | 66800 |
| 70-79 | 37300 |
| 80+ | 15450 |
| Total | 1000350 |

Reference: Ahmad OB, Boschi-pinto C, Lopez AD. Age standardization of rates: a new WHO standard. GPE Discuss Pap Ser. 2001;(31):1-14.
