# Supplementary material for: Population-based incidence rates and increased risk of EGFR mutated non-small cell lung cancer in Māori and Pacifica in New Zealand
Source: PLoS One. 2021 May 7;16(5):e0251357. doi: 10.1371/journal.pone.0251357 (PMC8104366; doi:10.1371/journal.pone.0251357)
Supplement: S2 Table — (DOCX) [file pone.0251357.s004.docx]

Table S2. Numbers of resident population, shown by smoking status, based on 2013 New Zealand census data.

|  |  | Total | Never-smoker | Ever-smoker |
| --- | --- | --- | --- | --- |
|  |  | N | N (%) | N (%) |
| Overall | | 1250028 | 746328 (59.7) | 378318 (30.3) |
| Age | | |  |  |
|  | 15-29 | 336897 | 230127 (68.3) | 68895 (20.4) |
|  | 30-39 | 210309 | 123930 (58.9) | 66669 (31.7) |
|  | 40-49 | 231687 | 133851 (57.8) | 76494 (33.0) |
|  | 50-59 | 198786 | 108414 (54.5) | 71340 (35.9) |
|  | 60-69 | 144297 | 77415 (53.6) | 52977 (36.7) |
|  | 70-79 | 81291 | 45570 (56.1) | 27864 (34.3) |
|  | 80+ | 46761 | 27021 (57.8) | 14079 (30.1) |
| Gender | |  |  |  |
|  | Male | 598452 | 335676 (56.1) | 201441 (33.7) |
|  | Female | 651573 | 410646 (63.0) | 176889 (27.1) |
| Ethnicity | |  |  |  |
|  | NZ European | 724995 | 435231 (60.0) | 264264 (36.5) |
|  | Māori | 126414 | 54657 (43.2) | 64890 (51.3) |
|  | Pacific | 129879 | 79032 (60.9) | 41811 (32.2) |
|  | Asian | 249759 | 202134 (80.9) | 34779 (13.9) |

Reference: Statistics New Zealand. Cigarette smoking behaviour and ethnic group (detailed total responses) by age group and sex, for the census usually resident population count aged 15 years and over, 2006 and 2013 Censuses (DHB areas). NZ.Stat Get data on demand. http://nzdotstat.stats.govt.nz/wbos/Index.aspx?DataSetCode=TABLECODE8292. Published 2013. Accessed April 22, 2020.
