# Supplementary material for: Population-based incidence rates and increased risk of EGFR mutated non-small cell lung cancer in Māori and Pacifica in New Zealand
Source: PLoS One. 2021 May 7;16(5):e0251357. doi: 10.1371/journal.pone.0251357 (PMC8104366; doi:10.1371/journal.pone.0251357)
Supplement: S3 Table — (DOCX) [file pone.0251357.s005.docx]

Table S3. Different types of EGFR mutation by numbers of patients

| Type of EGFR mutation | | Number | Percent |
| --- | --- | --- | --- |
| Total | | 384 | 100.0 |
|  | Exon 19 deletion | 170 | 44.3 |
|  | Exon 21 L858R | 139 | 36.2 |
|  | Exon 20 insertion | 33 | 8.6 |
|  | Exon 18 G719X | 15 | 3.9 |
|  | Exon 18 G719X + Exon 20 S768I | 10 | 2.6 |
|  | Exon 20 S768I | 3 | 0.8 |
|  | Exon 20 S768I + Exon 21 L858R | 3 | 0.8 |
|  | Exon 18 G719X + Exon 18 E709A | 2 | 0.5 |
|  | Exon 21 L858R + Exon 20 T790M | 2 | 0.5 |
|  | Exon 21 L861Q | 2 | 0.5 |
|  | Exon 20 R776C + Exon 21 L858R | 1 | 0.3 |
|  | Exon 18 G719X + Exon 21 L861Q | 1 | 0.3 |
|  | Exon 19 deletion + Exon 20 S768I | 1 | 0.3 |
|  | Exon 19 deletion + Exon 20 T790M | 1 | 0.3 |
|  | Exon 20 T790M | 1 | 0.3 |
